# Artemisinin: A Promising Adjunct for Cancer Therapy

**DOI:** 10.7759/cureus.3628

**Published:** 2018-11-23

**Authors:** Yamna Waseem, Choudhary A Hasan, Furqan Ahmed

**Affiliations:** 1 Internal Medicine, Dow University of Health Sciences (DUHS), Karachi, PAK; 2 Surgery, Dow University of Health Sciences (DUHS), Karachi, PAK

**Keywords:** artmesinin, cancer, malaria

## Abstract

Artemisinin is a herb derived from Artemesia annua (also known as sweet wormwood) and is known for its use as effective antimalarial pharmacotherapy. Recent studies have shown that artemisinin has anti-angiogenic and growth inhibition effects, as well as an apoptotic ability secondary to its inherent endo-peroxidase activity.

## Editorial

Cancer is a leading cause of non-communicable deaths in Pakistan. With an aging population, the burden of the disease is expected to upsurge, making cancer a potentially significant health burden and adding to the burden of diseases in a developing country like Pakistan [1]. Even though recent advancements in oncological therapies have shown great promise, these carry with them a massive healthcare cost. As a result, these therapies showcase limited use in a low-income country, where the majority of the patients bear their own medical expenses.

It is essential that the mode of therapy employed in cancer treatment has favorable potency, efficacy, and little to no side effects on the normal cells of the body. Although advanced medical care for cancer treatment promises improved survival rates and complete cure, the burden of adverse effects on quality of life has led to hesitancy and patient disapproval for various cancer care modalities. Until recently, a Chinese medicinal herb traditionally called qing hao or sweet wormwood, which has artemisinin, a lactone component of the Artemisia annua plant, as its active ingredient has shown promising results as an anticancer pharmacological intervention. The compound artemisinin has been used actively as a potent antimalarial drug, as well as boost the immune system and treat liver conditions [2]. Further research on this compound revealed that the anticancer mechanism of artemisinin is similar to its antimalarial mechanism, thereby being activated by heme, an iron-containing compound. Artemisinin has an endoperoxide moiety that forms free radicals when it reacts with iron, and the formation of free radicals mediates cellular damage and apoptosis of cells containing abnormally high levels of iron. Due to rapid cell division and the high metabolism rate of cancer cells, the high levels of iron intake constitute artemisinin as a targeted therapy for cancer and make cancer cells more susceptible to the cytotoxic effects of the compound [3]. A specific dosage for maximum efficacy is yet to be established; however, as a general principle, 400 to 800 mg per day can be used for at least six to 12 months, with no apparent adverse effects [2].

Artemisinin is invariably a valuable compound to combat cancer and its effectiveness is enhanced by the fact that is it effective orally and is cheap, as compared to other pharmacological interventions available on the market. It produces fewer side-effects, which makes it a favorable option for treatment in low-income settings where cancer has deep roots, mainly because of expensive treatments and the reluctance to opt for traditional regimens for cancer [3]. Furthermore, a recent study conducted by Lin Qingsong et al. found that the addition of aminolevulinic acid (ALA) enhances the anticancer properties of artemisinin against colorectal cancer cell lines [4]. Thus, if a purposeful plan is devised to develop artemisinin compounds as an adjunct to use in cancer treatments, it will contribute to decreasing financial costs for medical therapies. Given that more than 25 species of artemisinin are found in Pakistan [5], oncologists in Pakistan should take advantage of this drug and explore its benefits in the treatment of various cancers.
